# Spatial-dependent suppressive aftereffect produced by a sound in the rat’s inferior colliculus is partially dependent on local inhibition

**DOI:** 10.3389/fnins.2023.1130892

**Published:** 2023-03-20

**Authors:** Syed Anam Asim, Sarah Tran, Nicholas Reynolds, Olivia Sauve, Huiming Zhang

**Affiliations:** Department of Biomedical Sciences, University of Windsor, Windsor, ON, Canada

**Keywords:** binaural hearing, inferior colliculus, GABAergic inhibition, glycinergic inhibition, forward masking, free-field stimulation, suppressive aftereffect, sound location

## Abstract

In a natural acoustic environment, a preceding sound can suppress the perception of a succeeding sound which can lead to auditory phenomena such as forward masking and the precedence effect. The degree of suppression is dependent on the relationship between the sounds in sound quality, timing, and location. Correlates of such phenomena exist in sound-elicited activities of neurons in hearing-related brain structures. The present study recorded responses to pairs of leading-trailing sounds from ensembles of neurons in the rat’s inferior colliculus. Results indicated that a leading sound produced a suppressive aftereffect on the response to a trailing sound when the two sounds were colocalized at the ear contralateral to the site of recording (i.e., the ear that drives excitatory inputs to the inferior colliculus). The degree of suppression was reduced when the time gap between the two sounds was increased or when the leading sound was relocated to an azimuth at or close to the ipsilateral ear. Local blockage of the type-A γ-aminobutyric acid receptor partially reduced the suppressive aftereffect when a leading sound was at the contralateral ear but not at the ipsilateral ear. Local blockage of the glycine receptor partially reduced the suppressive aftereffect regardless of the location of the leading sound. Results suggest that a sound-elicited suppressive aftereffect in the inferior colliculus is partly dependent on local interaction between excitatory and inhibitory inputs which likely involves those from brainstem structures such as the superior paraolivary nucleus. These results are important for understanding neural mechanisms underlying hearing in a multiple-sound environment.

## Introduction

A natural acoustic environment typically contains multiple qualitatively different sounds that are generated at different time and locations. The perception of one sound can be affected by another sound, with the effect being dependent on the temporal and spatial relationships as well as the qualitative difference between the sounds (Bregman, 1990; Feng and Ratnam, 2000). For instance, a speech sound can be masked by an interfering sound in a temporospatial-dependent manner (Cherry, 1953; Bronkhorst, 2000; Shinn-Cunningham et al., 2001; Arbogast et al., 2002; Jones and Litovsky, 2011; Warzybok et al., 2013; Yost, 2017).

Responses to multiple sounds have been recorded in brain structures to study neural bases of auditory perception in a natural acoustic environment. In the inferior colliculus (IC, a midbrain nucleus), neurons display two-tone suppression when two tone bursts are simultaneously presented (Zhang and Feng, 1998; Egorova et al., 2001; Alkhatib et al., 2006; Hurley et al., 2008). Action-potential firing elicited by a tone burst at a neuron’s characteristic frequency (CF, the frequency at which the threshold of response is the lowest) is suppressed by another tone burst at a different frequency. When two sounds are temporally separated, the response to a trailing sound can be suppressed by a leading sound with the degree of suppression being dependent on the time gap between the sounds (Finlayson and Adam, 1997; Faure et al., 2003; Mei et al., 2006; Nelson et al., 2009; Zhang and Kelly, 2009; Singheiser et al., 2012; Gai, 2016). When two simultaneously presented sounds are spatially separated, the response to a sound with a fixed location can be suppressed by a relocated sound, with the effect being dependent on the angle of separation (Ratnam and Feng, 1998; Lane and Delgutte, 2005; Day et al., 2012). A suppressive effect can be observed even when two sounds are separated both temporally and spatially (Yin, 1994; Litovsky and Yin, 1998a,b; Tolnai et al., 2017; Chot et al., 2019, 2020). Knowledge about the neurobiological bases of such suppressive effects can greatly help us understand hearing in a natural acoustic environment.

The strength of the sound-driven response of an auditory neuron is dependent on excitatory/inhibitory inputs received by the neuron. For most IC neurons, major excitatory inputs are from the contralateral cochlear nucleus and lateral superior olivary nucleus (LSO), and the ipsilateral medial superior olivary nucleus (Loftus et al., 2004; Cant, 2005). Major inhibitory inputs are from the contralateral dorsal nucleus of the lateral lemniscus (DNLL) and the ipsilateral LSO, superior paraolivary nucleus (SPN), and dorsal as well as ventral nuclei of the lateral lemniscus (VNLL) (Helfert et al., 1989; Saint Marie et al., 1989; González-Hernández et al., 1996; Zhang et al., 1998; Kulesza and Berrebi, 2000; Loftus et al., 2004; Saldaña et al., 2009).

The dependence of sound-driven responses of IC neurons on excitatory/inhibitory inputs is supported by neurophysiological results (Xie et al., 2008; Liu et al., 2019). Characteristics of responses such as two-tone suppression can be affected by local blockage of inhibition (Hurley et al., 2008). When a sound that is presented in a free acoustic field is relocated from the contralateral to the ipsilateral ear, many neurons reduce firing over the duration of the sound due to decreased excitation and/or increased inhibition (Park and Pollak, 1994; Zhang et al., 1999; Poirier et al., 2003). In case another sound with a fixed location is presented simultaneously, such changes in excitation and/or inhibition can conceivably affect the responses of neurons to the second sound. A sound can activate inhibitory inputs to IC neurons even when the sound is presented at the contralateral ear. Such inhibitory inputs can be provided by the ipsilateral SPN (Saldaña et al., 2009). As neurons in this structure receive inputs from the contralateral cochlear nucleus and fire action potentials at the offset of a sound, outputs from these neurons to the IC can allow a contralaterally presented sound to suppress responses of IC neurons to a subsequent sound (Schofield, 1995; Kulesza et al., 2003; Kadner and Berrebi, 2008; Felix et al., 2015, 2017; Gai, 2016; Salimi et al., 2017). An inhibitory aftereffect can also be generated on IC neurons through the activation of neurons in the dorsal nucleus of the lateral lemniscus (Burger and Pollak, 2001; Pecka et al., 2007). Further studies have yet to be conducted to characterize the temporal and spatial dependences of the suppressive aftereffect produced by a sound on IC neurons.

In the present study, we used a pair of leading and trailing sounds to elicit local-field potentials (LFPs) in the IC. We studied how the suppressive aftereffect produced by a leading sound was dependent on the temporal and spatial separations between the two sounds. We used pharmacological agents to block GABA_*A*_ and glycine receptors in the IC to study how local inhibition contributed to the suppressive aftereffect.

## Materials and methods

### Animal preparation

Eighteen adult male Wistar albino rats (*Rattus norvegicus*) were obtained from Charles River Canada Inc. (St. Constant, QC, Canada). They were 210–600 g when experiments were conducted. Surgical anesthesia was induced by ketamine hydrochloride (60 mg/kg, i.m.) and xylazine hydrochloride (10 mg/kg, i.m.) and maintained by ketamine hydrochloride (20 mg/kg, i.m.) and xylazine hydrochloride (3.3 mg/kg, i.m.).

A craniotomy was made in the skull over the right temporal lobe for placing a recording electrode into the IC. The skull was cemented onto a head bar attached to a custom-made holding instrument. A recording electrode was held by a custom-made clamp attached to the slave cylinder of a Model 650 micropositioner, which was fitted onto a Model 900 stereotaxic instrument (Kopf Instruments, Tujunga, CA, USA). The rat, along with the head-holding and electrode positioning instruments, was placed in a Model CL-15A LP acoustic chamber (Eckel Industries, Morrisburg, ON, Canada) when sound-driven responses were recorded. Instruments were positioned in such a way that acoustic shadows and reflections were minimized. All procedures were approved by the University of Windsor Animal Care Committee in accordance with the guidelines of the Canadian Council on Animal Care.

### Acoustic stimulation

**FIGURE 1:**
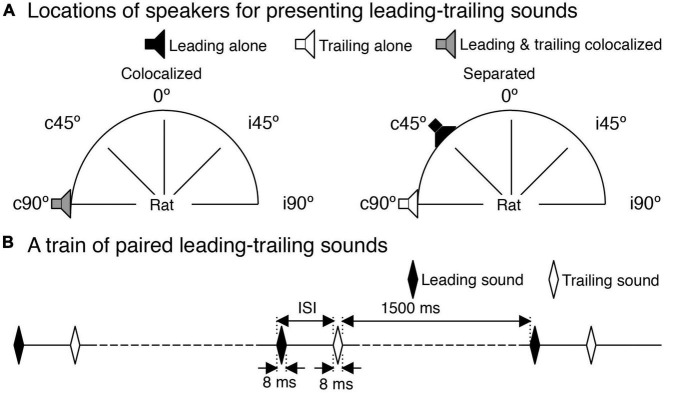
Speaker locations and a train of paired leading-trailing sounds. **(A)** Five azimuthal locations (c90°, c45°, 0°, i45°, and i90°) used in sound presentations. A pair of leading-trailing tone bursts were either colocalized at c90° (left panel) or separated with a leading sound being at a non-c90° azimuth while a trailing sound being at c90° (right panel for a leading sound at c45°). Two speakers used in the study were calibrated at these azimuths. **(B)** A train of leading-trailing tone-burst pairs. In panels **(A,B)**, a leading sound or a speaker that was used to present a leading sound is indicated by a black color. A trailing sound or a speaker that was used to present a trailing sound is indicated by a white color. A speaker that was used to present both a leading and a trailing sound (colocalized at c90°) is indicated by a gray color.

Sound waveforms were generated using a System 3 real-time signal processing system controlled by a personal computer running the OpenEx software (Tucker-Davis Technologies, Alachua, FL, USA). Sounds were presented using two FF1 free-field speakers (Tucker-Davis Technologies, Alachua, FL, USA) that could be positioned at any azimuthal location that was 50 cm away from the midpoint of the interaural line of a rat. Each speaker was calibrated over the range between 100 and 40,000 Hz using a model 2608 measuring amplifier and a model 4135 microphone (Brüel & Kjaer, Dorval, QC, Canada). The microphone was positioned at the location where the midpoint of the interaural line would be. Calibration was conducted with the speaker at each of the five locations used in the present study, which included the midline of the frontal field and 90° and 45° on the contra- and ipsilateral side of the recording site (Figure 1A, denoted by 0°, c90°, c45°, i45°, and i90°).

### Recording electrode and procedures

A “piggy-back” multi-barrel electrode assembly was used to record an LFP from an ensemble of neurons in the IC and to release neuropharmacological agents at the site of recording (Patel and Zhang, 2014). The electrode assembly consisted of a single-barrel recording glass pipette (filled with 0.5 M sodium acetate with 3% Chicago Sky Blue, tip diameter ∼15 μm, impedance 100–300 kΩ) and a 5-barrel “H-configuration” drug-release glass pipette (tip diameter 15–20 μm). The tip of the single-barrel pipette protruded beyond the tip of the multi-barrel pipette by 20–25 μm. Each of the side barrels of the 5-barrel pipette was filled with the GABA_*A*_ receptor antagonist Gabazine or the glycine receptor antagonist strychnine (both 5 mM in physiological saline, pH 3.5). The central barrel was filled with physiological saline for the balance of electrical current.

Signals registered by the recording pipette were amplified by 1,000 times and bandpass filtered (0.3–300 Hz) by a model 2,400A preamplifier (Dagan, Minneapolis, MN, USA). The signals were sampled at 3.1 kHz using the System 3 real-time signal processing system. The five barrels of the drug-release electrode were connected to a BH-2 Neurophore microiontophoretic system (Harvard Apparatus, Holliston, MA, USA). To prevent drug leakage, a current around −20 nA was applied to each of the side barrels when a pharmacological agent was not being released.

An electrode assembly was inserted into the right IC while Gaussian noise bursts at 60 dB SPL were presented from a loudspeaker at c90° to search for a site of recording. Recordings were conducted only on one side of the IC to minimize the utilization of electrode holding devices and avoid repositioning of holding devices, which helped maintain the consistency of an acoustic field. Upon identification of a recording site, the CF and the threshold at CF were determined for the site using tone bursts presented at c90°. These tone bursts had an 8-ms duration (4-ms rising/falling phases, no plateau).

A train of leading-trailing tone-burst pairs (see Figure 1B) was created for the recording site using two 8-ms tone bursts. A leading sound was randomly chosen from two tone bursts named T_*L*_ and T_*H*_. The frequencies of T_*L*_ and T_*H*_ were *f*_*L*_ (lower than CF) and *f*_*H*_ (higher than CF) and were calculated based on the CF of the recording site. The center frequency of *f*_*L*_ and *f*_*H*_ [i.e., (*f*_*L*_ × *f_*H*_*)^1/2^] was at CF while the frequency difference between *f*_*L*_ and *f*_*H*_ was 10% of the center frequency [i.e., (*f*_*H*_ − *f*_*L*_)/(*f*_*H*_ × *f*_*L*_)^1/2^ = 0.1]. A trailing sound was a tone burst with a frequency at the CF of the recording site. The leading and trailing sounds had the same intensity, which was typically 30 dB above the threshold at CF. The interval between onsets of the two sounds (i.e., inter-stimulus interval or ISI) could be varied systematically. Each train had 60 pairs of leading-trailing tone bursts. The interval between the offset of a trailing sound of one pair and the onset of a leading sound of a subsequent pair was 1,500 ms.

Responses to a train of leading-trailing sound pairs were first recorded when the two sounds were colocalized at c90° (Figure 1A left panel). The ISI between leading and trailing sounds was systematically changed to examine how the suppressive effect of the leading sound was dependent on the temporal separation between the two sounds (i.e., the time course of the aftereffect). Recordings were then conducted when the leading sound was at other azimuths (i.e., i90°, c45°, i45°, and 0°) while the trailing sound remained at c90° to examine how the suppressive effect of the leading sound was dependent on the spatial separation between the sounds (see Figure 1A right panel for leading sound at c45°). At each angle of separation, ISI between the leading and trailing sounds was systemically varied. Sounds that were used to create a train of leading-trailing pairs were presented individually to elicit responses. These responses were used as references to evaluate whether/how the leading and training sounds in a train of sound pairs affected each other in eliciting responses. For this purpose, the response to a leading sound was recorded when the sound was at each of the five azimuths (i.e., c90°, i90°, c45°, i45°, and 0°) and the response to a trailing sound was recorded when the sound was at c90°.

Gabazine or strychnine was released microiontophoretically (current of injection at +3∼+140 nA) at the recording site to study whether/how the suppressive effect generated by a leading sound was dependent on local inhibition in the IC. The effect of a pharmacological agent was monitored by repeatedly recording responses to a leading and/or a trailing sound presented individually at c90°. For this purpose, a tone burst was presented at multiple (typically 7) intensities ranging from slightly below the threshold at the CF to well above the threshold (including the intensity used for creating a sound pair). At each intensity a tone burst was presented 20 times; and all presentations (i.e., 140 presentations for 7 intensities) had a random order. The rate of sound presentation was 1/sec. Responses elicited at different intensities were used to create an amplitude-intensity function (AIF, see data analysis for more details) and a latency-intensity function. When the effect of a drug on responses to individual sounds reached a stabilized level, responses to a train of sound pairs were recorded at various ISIs and angles of separation. AIFs for responses to the leading and the trailing sounds were also recorded. Following the evaluation of the effect of a drug, the injection current was terminated and a retention current was reapplied. Recovery was monitored by repeatedly recording responses to a leading or a trailing sound presented individually at c90°.

### Data analysis

**FIGURE 2:**
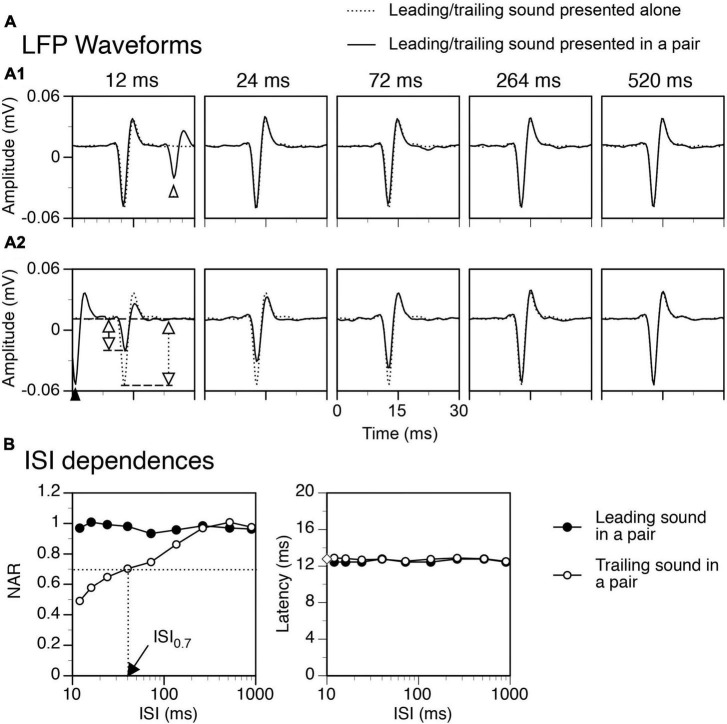
An example showing that a temporal separation between a leading and a trailing tone burst reduced the suppressive aftereffect produced by the leading sound. Results were obtained when the two sounds were colocalized at c90°. **(A)** Waveforms of local-field potentials (LFPs) elicited by a leading sound [solid lines in **(A1)**] and a trailing sound [solid lines in **(A2)**] in a leading-trailing sound pair. The waveforms shown in two corresponding panels in **(A1)** and **(A2)** were obtained at the same inter-stimulus interval (ISI) [indicated above a panel in **(A1)**]. The start of the x axis (0 ms) corresponds to the onset of a leading sound in each panel of **(A1)** and the onset of a trailing sound in each panel of **(A2)**. A part of the LFP elicited by a trailing sound is shown in the left panel of **(A1)** (pointed by an open upward triangle), while a part of the LFP elicited by a leading sound is shown in the left panel of **(A2)** (pointed by a filled upward triangle). LFPs elicited by the leading and the trailing sound when they were presented individually are shown in **(A1)** and **(A2)** (dotted lines) for comparison. In the left panel of **(A2)**, two vertical double arrows indicate amplitudes of the negative peak of LFP elicited by a trailing sound presented individually (dotted) and in a sound pair (solid). **(B)** Dependences of normalized amplitude of response (NAR, left panel) and latency (right panel) on the ISI for LFPs elicited by a leading (filled circle) and a trailing (open circle) sound. In the left panel, a horizontal dotted line indicates the level of NAR at 0.7. The vertical dotted line indicates the ISI associated with the 0.7 NAR. In the right panel, an open diamond on the Y-axis indicates the latency of the response to a trailing sound presented alone. The characteristic frequency (CF) of the recording site (hence the frequency of the trailing sound) was 13.0 kHz. The frequency of the leading sound was 13.458 kHz.

Neural signals collected over presentations of leading and trailing sounds in a train of paired stimuli were averaged separately to obtain two mean waveforms for LFPs elicited by the two sounds, respectively. Signals collected over presentations of a single tone burst at multiple intensities (see the last paragraph of “*Recording electrode and procedures*”) were averaged separately to obtain mean waveforms for LFPs elicited at these intensities, respectively. In agreement with our previous result (Patel et al., 2012), an LFP had a dominant negative peak that was preceded by a small positive peak and followed by an intermediate positive peak (Figure 2A). The amplitude and latency of the negative peak of an LFP were measured.

We evaluated whether the response to a sound (either leading or trailing) in a sound pair was affected by the other sound in the pair and how the response was dependent on the spatial location of a leading sound as well as the temporal separation between leading and trailing sounds. An index of the normalized amplitude of response (NAR) was calculated for these purposes:


$$N\,A\,R=A_{p\,a\,i\,r\,e\,d}\,\left(\theta ,\Delta \,t\right)/A_{a\,l\,o\,n\,e}\,\left(c\,90^{\circ }\right)$$

Where A_*paired*_ (θ, Δt) is the amplitude of the response to the sound under evaluation obtained when the sound was presented in a sound pair, with the leading and trailing sounds being at azimuth θ and c90°, respectively, and the interval between the two sounds being at Δt. A_*alone*_ (c90°) is the amplitude of the response to the sound under evaluation obtained when the sound was presented alone at c90°. A NAR value of 0 indicates that the response to a sound was completely suppressed in comparison to the response to the same sound presented alone at c90°, while a NAR value of 1 indicates that the response to a sound was not affected.

To determine whether a pharmacological agent affected local inhibition at the site of recording, an AIF was created using amplitudes of responses elicited by a single sound presented at a single azimuth but various intensities (see the first paragraph of “*Data analysis”*). AIFs obtained before and during the application of the drug were compared. When a change in AIF produced by a drug stabilized, NAR values were calculated for responses elicited by leading and trailing sounds in a sound pair at each combination of temporal and angular separations. NAR values for responses to a trailing sound obtained before and during drug application were compared to find how the drug influenced a suppressive aftereffect.

Statistical analysis was conducted using the SPSS (version 23) software (IBM Corporation, Armonk, NY, USA).

## Results

LFPs elicited by single tone bursts and pairs of leading-trailing tone bursts were recorded in the right IC in each of the 18 rats used in the present study. The rats formed two groups, with T_*L*_ presented as a leading sound in one group (*n* = 9) while T_*H*_ presented as a leading sound in another group (*n* = 9). Data obtained from the two groups were not different from each other (not shown). Thus, the data are combined in this article.

### Response to a leading sound was not affected by a trailing sound over a train of sound pairs

**FIGURE 3:**
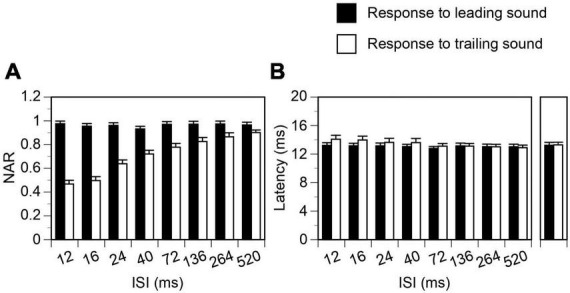
Group results showing that temporal separation between a leading and a trailing tone burst reduced the suppression of the response to a trailing sound but did not affect the response to a leading sound. Results were obtained when leading and trailing sounds were colocalized at c90°. **(A,B)** Show effects of separation on normalized amplitude of responses (NARs) and latencies of local-field potentials (LFPs), respectively. Latencies of LFPs elicited by a leading and a trailing sound when they were presented alone at c90° are shown on the right of panel **(B)**. An error bar indicates a standard error of the mean.

Shown in Figure 2 are LFPs obtained from the IC of an example rat when a leading and a trailing sound were colocalized at c90°. Regardless of the ISI between the two sounds, the waveform (including amplitude and latency) of the LFP elicited by a leading sound was very similar to that of the LFP elicited by the same sound presented individually at c90° (Figure 2A1). This similarity was observed at all combinations of ISI and angle of separation between the two sounds and supported by group results from 18 animals. Shown in Figure 3 are group results obtained when two sounds were colocalized at c90° (black bars for responses to a leading sound, One-way ANOVA, *F* = 0.410, *p* = 0.894 for amplitude, and *F* = 0.111, *p* = 0.998 for latency, respectively). Thus, over a train of leading-trailing sound pairs the response to a leading sound was not affected by preceding presentations of a trailing sound.

### Suppression of the response to a trailing sound by a colocalized leading sound

The response to a trailing tone burst at c90° was greatly suppressed by a colocalized leading tone burst when the two sounds were separated by a small ISI (see Figure 2A2 left panel for an example). The degree of suppression was reduced when the ISI was increased (Figure 2A2 panels 2–5 and Figure 2B left panel). The NAR value was 0.7 (i.e., the amplitude of the response was suppressed by 30%) when ISI was close to 40 ms (Figure 2B left panel). Such an ISI value, namely ISI_0.7_, was used in the article to reflect the duration of suppression. When the ISI was 264 ms, the response to a trailing sound was almost identical to that elicited by the sound presented alone. The latency of the response to a trailing sound was not affected by a leading sound at any ISIs (Figures 2A2, B right panel). Results from the entire group of animals confirmed that when leading and trailing sounds were colocalized at c90° the amplitude of the response to a trailing sound was dependent on the ISI while the latency of the response was not (Figure 3 white bars, One-way ANOVA, *F* = 29.168, *p* < 0.001 for amplitude and *F* = 1.084, *p* = 0.377 for latency).

### Relocation of a leading sound changed both responses to leading and trailing sounds

**FIGURE 4:**
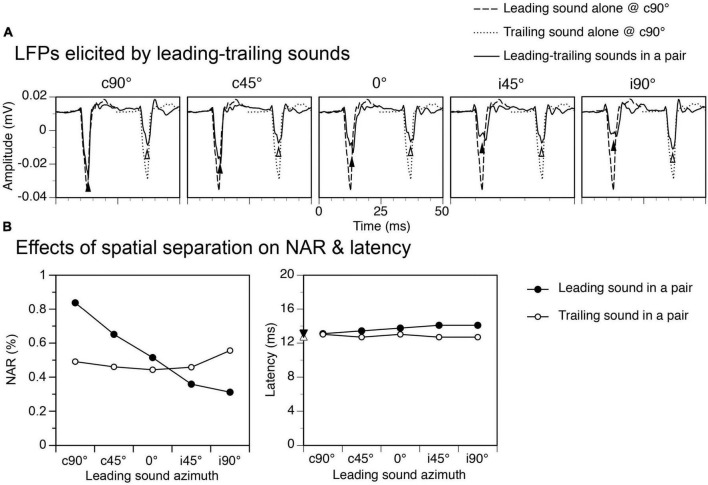
An example showing effects of spatial separation between a leading and a trailing tone burst on the local-field potentials (LFPs) elicited by the sounds. The time interval between leading and trailing tone bursts was 24 ms. **(A)** Waveforms of LFPs elicited by leading and trailing tone bursts. Results were obtained when a leading tone burst was at c90°, c45°, 0°, i45°, and i90° (indicated above each panel) while a trailing tone burst was at a fixed location at c90°. The start of the x axis (0 ms) corresponds to the onset of a leading sound in each panel of **(A)**. Filled and open upward triangles indicate LFPs elicited by the leading and the trailing sound, respectively. Waveforms of LFPs elicited by the leading and the trailing tone burst presented alone at c90° are shown in each panel for comparison. **(B)** Line charts showing effects of spatial separation between leading and trailing sounds on normalized amplitude of responses (NARs) (left panel) and latencies (right panel) of LFPs elicited by the sounds. Filled downward and open upward triangles on the y-axis of the right panel indicate the latencies of the responses elicited by a leading and a trailing sound alone at c90°, respectively. The characteristic frequency (CF) of the recording site (hence the frequency of the trailing sound) was 5.5 kHz. The frequency of the leading sound was 5.694 kHz.

Relocating a leading sound from c90° to another azimuth reduced the amplitude of the response to the sound, as shown by an example in Figure 4 (Figure 4A and the left panel of Figure 4B, results obtained at an ISI at 24 ms). Such a reduction was not caused by presentations of a trailing sound in a train of sound pairs (see first subsection of “Results”). Rather, it reflected a direction-dependence of the response to the leading sound. A reduction of the response to a leading sound was accompanied by a reduction of suppression of the response to a trailing sound, which remained at c90°. The NAR value for the response was slightly below 0.50 when the leading sound was at c90° but above 0.55 when the leading sound was at i90°. For both responses to the leading and trailing sounds, the latency was minimally affected by the relocation of a leading sound (Figure 4B right panel).

**FIGURE 5:**
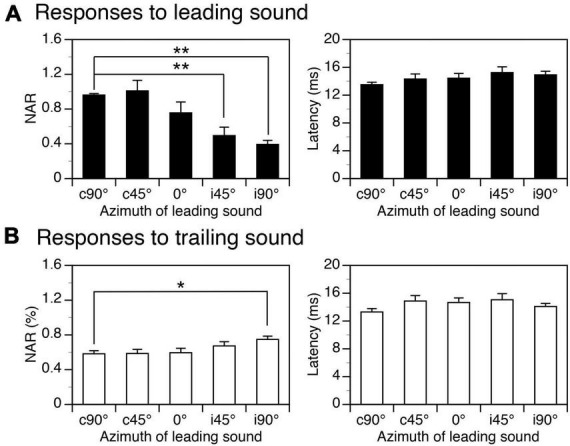
Group results showing effects of spatial separation between a leading and a trailing tone burst on the amplitudes and latencies of local-field potentials (LFPs) elicited by the sounds. **(A,B)** Bar charts based on responses to a leading and a trailing sound, respectively. In both panels **(A,B)**, the left and right panels show effects of spatial separation on the normalized amplitude of response (NAR) and the latency, respectively. An error bar indicates a standard error of the mean. “*” and “**” indicate statistical significance (Tukey’s test) at the level of 0.05 and 0.005, respectively.

Group results obtained when the ISI was at 24 ms confirmed that both NAR values for responses to a leading and a trailing sound were dependent on the location of the leading sound (Figures 5A, B left panels, One-way ANOVA, *F* = 13.917, *p* < 0.001 for response to leading sound, *F* = 3.384, *p* = 0.014 for response to trailing sound). The Tukey’s test revealed that the NAR value for the response to a leading sound was significantly reduced when the sound was relocated from c90° to i45° (*p* = 0.001) or i90° (*p* < 0.001). The same test revealed that the NAR value for the response to a trailing sound was significantly increased when the leading sound was relocated from c90° to i90° (*p* = 0.021). Latencies of responses to leading and trailing sounds were not changed by the relocation of a leading sound (One-way ANOVA, *F* = 1.464, *p* = 0.221 for Figure 5A right panel and *F* = 1.432, *p* = 0.233 for Figure 5B right panel).

**FIGURE 6:**
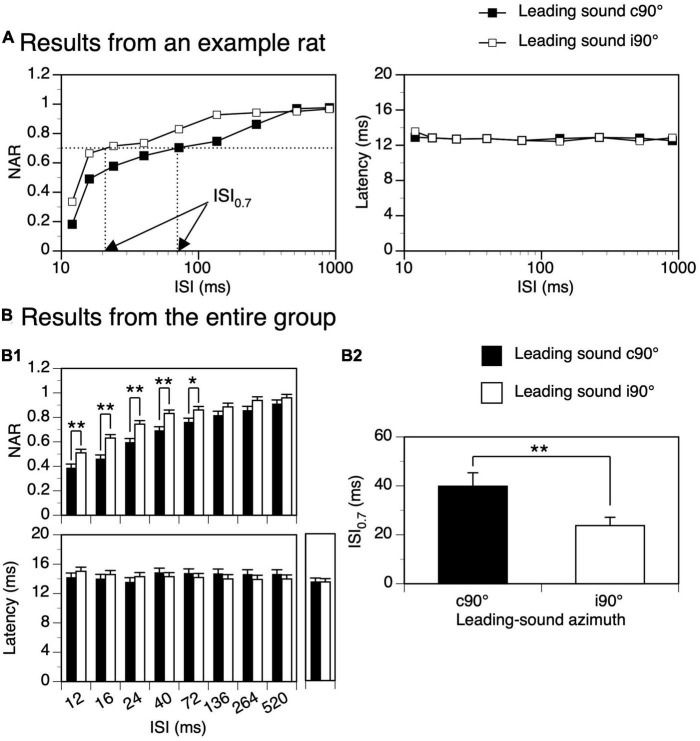
Difference between suppressive aftereffects produced by a leading tone burst presented at c90° and i90°. **(A)** Results from an example rat showing normalized amplitude of response (NAR)-inter-stimulus interval (ISI) (left panel) and latency-ISI (right panel) relationships for the response to a trailing sound obtained when the leading sound was at c90° and i90°, respectively. In the left panel, two arrows point toward the ISI_0.7_ values obtained when the leading sound was at the two azimuths. The characteristic frequency (CF) of the recording site (hence the frequency of the trailing sound) was 13.0 kHz. The frequency of the leading sound was 13.458 kHz. **(B)** Results from the entire group of 18 rats comparing effects generated by a leading sound at c90° and i90°. (B1) Top and bottom panels show effects of a leading sound on the NAR and latency of the response to a trailing sound, respectively. A *post-hoc t*-test (see text for the result from a two-way ANOVA test) indicates that the difference in the suppressive effect was significant at all ISIs between 12 and 72 ms (*p* = 0.004 at 12 ms; *p* < 0.001 at 16 ms; *p* < 0.001 at 24 ms; *p* = 0.001 at 40 ms; *p* = 0.020 at 72 ms). Before responses to a leading-trailing sound pair were recorded at each angle of separation (c90° and i90°), the response to a trailing sound presented alone was recorded. Latencies of responses elicited by a trailing sound under these conditions are shown on the right side of the bottom panel. (B2) Bar chart comparing ISI_0.7_ values obtained when the leading sound was at c90° and i90°. An error bar indicates a standard error of the mean. “*” and “**” indicate statistical significance at the level of 0.05 and 0.005, respectively.

Time courses of suppression produced by a leading sound at c90° and i90° were compared in each animal. Shown in Figure 6A left panel are NAR-ISI curves for responses to a trailing sound obtained from an example animal. The degree of suppression at short ISIs was higher and suppression lasted longer when the leading sound was at c90° than at i90°. In agreement with these differences, the ISI_0.7_ value was larger when the leading sound was at c90° than at i90°. Group results from 18 animals supported the difference between the suppressive effects produced by a leading sound at c90° and at i90° (Figure 6B1 top panel, two-way ANOVA, *F* = 53.686, *p* < 0.001). A *post-hoc t*-test indicated that the difference was significant at all ISIs between 12 and 72 ms (see Figure 6 caption for statistical results). The ISI_0.7_ value was larger when the sound was at c90° than i90° (Figure 6B2, Paired *t*-test, *t* = 3.230, *p* = 0.004). The latency of the response to a trailing sound was not affected by the ISI and the location of a leading sound (see Figure 6A right panel for an example and Figure 6B1 bottom panel for group results, two-way ANOVA, *F* = 0.365, *p* = 0.546).

### Effects of pharmacological manipulations

**FIGURE 7:**
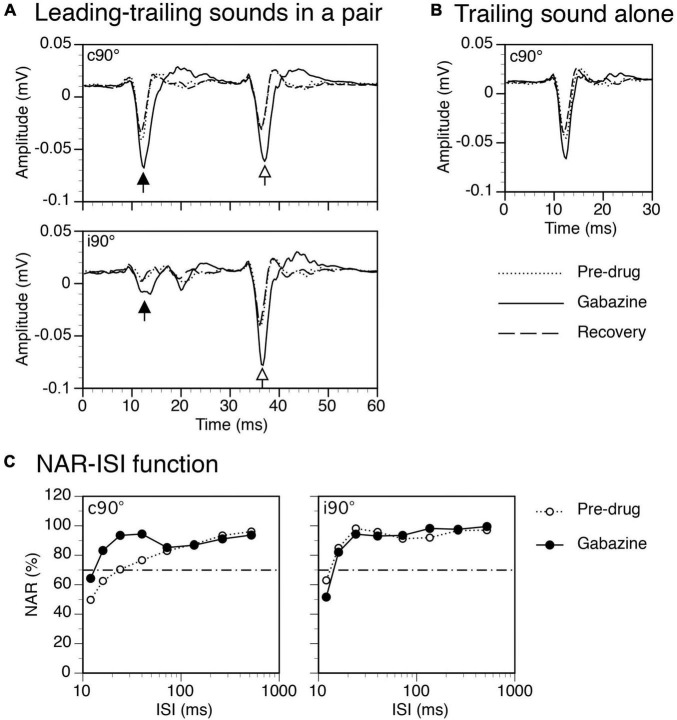
Results from an example rat showing that gabazine enhanced local-field potentials (LFPs) elicited by a leading and a trailing sound and reduced the suppressive aftereffect produced by a leading sound. **(A)** The effect of gabazine on waveforms of LFPs elicited by a leading (indicated by a filled upward arrowhead) and a trailing sound (indicated by an open upward arrowhead) in a pair (ISI = 24 ms). Results were obtained when the leading sound was presented at c90° (top panel) and i90° (bottom panel), respectively. **(B)** The effect of gabazine on the waveform of an LFP elicited by the trailing sound presented alone at c90°. **(C)** The effect of gabazine on the normalized amplitude of response (NAR)-ISI relationship obtained when the leading sound was at c90° (left panel) and i90° (right panel), respectively. The horizontal dash-and-dotted line indicates the value of NAR at 0.7. The characteristic frequency (CF) of the recording site (hence the frequency of the trailing sound) was 12.0 kHz. The frequency of the leading sound was 11.501 kHz.

Gabazine released at the site of recording enhanced both LFPs elicited by a leading and a trailing sound when they were presented in a pair (Figure 7A). It also enhanced responses to the sounds when they were presented individually (Figure 7B for response to a trailing sound). For the example shown in Figure 7, the suppressive aftereffect produced by a leading sound was reduced (i.e., the NAR value for the response to a trailing sound was increased) by gabazine at short ISIs when the leading sound was at c90° (Figure 7C left panel). The aftereffect was minimally changed by the drug when the leading sound was at i90° (Figure 7C right panel).

**FIGURE 8:**
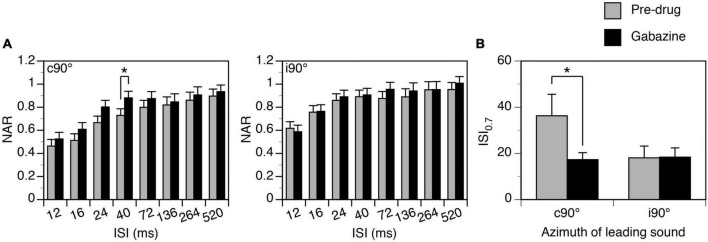
Group results showing that gabazine partially reduced the suppressive aftereffect produced by a leading sound. **(A)** Bar charts showing effects of gabazine on the normalized amplitude of response (NAR)-inter-stimulus interval (ISI) relationship. Results were obtained when a leading sound was presented at c90° (left panel) and i90° (right panel). The location of the leading sound is shown in the top left corner of each plot. **(B)** A bar chart showing the effect of gabazine on the ISI_0.7_ obtained at c90° and i90°. An error bar indicates a standard error of the mean. “*” indicates statistical significance at the level of 0.1.

Group results (*n* = 8) support that gabazine changed the degree of suppression when a leading sound was at c90° (Figure 8A left panel, Two-way ANOVA, *F* = 6.596, *p* = 0.012). A *post-hoc t*-test indicated that the reduction was significant at the level of *p* < 0.1 when the ISI was at 40 ms (*p* = 0.063). An increase of NAR value at short ISIs was accompanied by a decrease of ISI_0.7_ value at a statistical significance level at *p* < 0.1 (Figure 8B, Paired *t*-Test, *t* = 1.958, *p* = 0.091). When a leading sound was at i90°, gabazine did not change the degree of suppression (Figure 8A right panel, Two-way ANOVA, *F* = 1.078, *p* = 0.305). It did not change the ISI_70_, either (Paired *t*-test, *t* = 0.554, *p* = 0.604).

**FIGURE 9:**
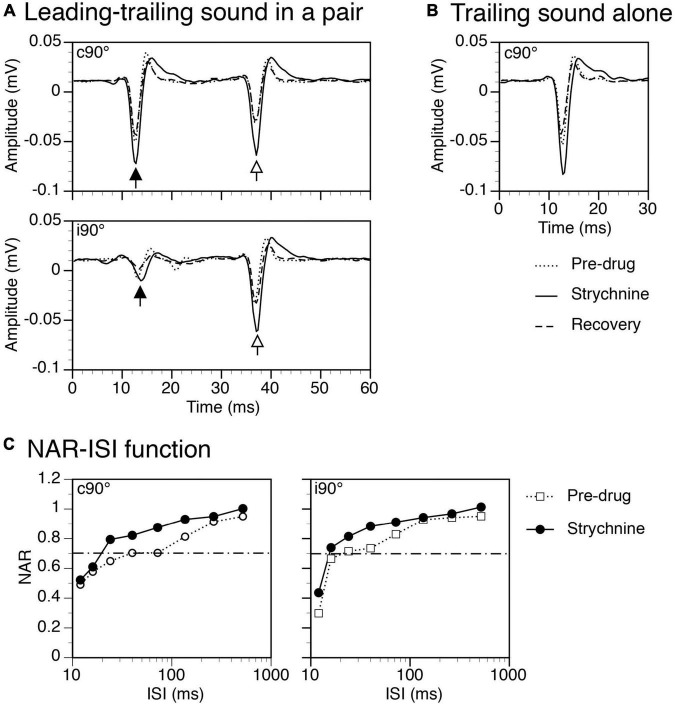
Results from an example rat showing that strychnine enhanced local-field potentials (LFPs) elicited by leading and trailing sounds and reduced the suppressive aftereffect produced by a leading sound. **(A)** The effect of strychnine on waveforms of LFPs elicited by a leading (indicated by a filled upward arrowhead) and a trailing sound (indicated by an open upward arrowhead) in a pair (ISI = 24 ms). Results were obtained when the leading sound was presented at c90° (top panel) and i90° (bottom panel), respectively. **(B)** The effect of strychnine on the waveform of an LFP elicited by the trailing sound presented alone at c90°. **(C)** The effect of strychnine on the normalized amplitude of response (NAR)-inter-stimulus interval (ISI) relationship obtained when the leading sound was at c90° (left panel) and i90° (right panel), respectively. The horizontal dash-and-dotted line indicates the value of NAR at 0.7. The characteristic frequency (CF) of the recording site (hence the frequency of the trailing sound) was 13.0 kHz. The frequency of the leading sound was 13.458 kHz.

Strychnine increased amplitudes of both responses to a leading and a trailing sound no matter whether the sounds were presented in a pair (Figure 9A) or individually (Figure 9B for the response to a trailing sound). NAR-ISI functions revealed that the suppressive effect of a leading sound was moderately reduced over a wide range of ISIs both when a leading sound was at c90° and at i90° (Figure 9C left and right panels).

**FIGURE 10:**
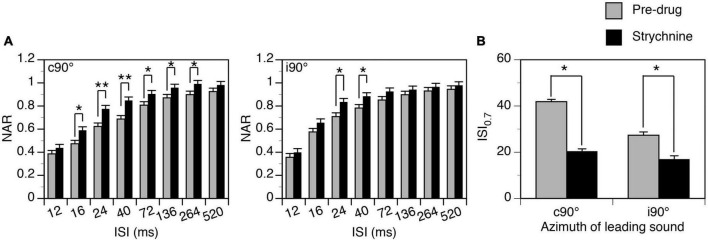
Group results showing that strychnine reduced the suppressive aftereffect produced by a leading sound at some inter-stimulus intervals (ISIs). **(A)** Bar charts showing the effect of glycine on the normalized amplitude of response (NAR)-ISI relationship. Results were obtained when a leading sound was presented at c90° (left panel) and i90° (right panel). **(B)** A bar chart showing the effect of glycine on the ISI_0.7_ obtained at c90° and i90°. A *post-hoc t*-test (see text for the result from a two-way ANOVA test) indicates that strychnine significantly changed the NAR values at ISIs at 16 ms (*p* = 0.007), 24 ms (*p* < 0.001), 40 ms (*p* < 0.001), 72 ms (*p* = 0.023), 136 ms (*p* = 0.039), and 264 ms (*p* = 0.030) when a leading sound was at c90°. The drug significantly changed the NAR values at ISIs at 24 ms (*p* = 0.005) and 40 ms (*p* = 0.018) when a leading sound was at i90°. An error bar indicates a standard error of the mean. “*” indicates statistical significance at the level of 0.05, while “**” indicates statistical significance at the level of 0.005.

Group results (*n* = 7) indicate that strychnine reduced the degree of suppression generated by a leading sound when the sound was at c90° (Two-way ANOVA, *F* = 48.492, *p* < 0.001) and when it was at i90° (Two-way ANOVA, *F* = 16.650, *p* < 0.001). A significant drug-induced change was observed at all ISIs between 16 and 264 ms when a leading sound was at c90° and at ISIs at 24 and 40 ms when a leading sound was at i90° (*Post-hoc t*-test, see Figure 10 caption for statistical results). A reduced degree of suppression was accompanied by a decreased ISI_0.7_ value when a leading sound was at c90° (Paired *t*-test, *t* = 3.475, *p* = 0.013) and i90° (*t* = 3.024, *p* = 0.023).

## Discussion

The present study revealed that a leading sound could produce an aftereffect to suppress an LFP elicited by a trailing sound in the IC. Such an aftereffect lasted for up to a few hundred milliseconds when the two sounds were co-localized at the ear contralateral to the site of recording. The degree and duration of suppression were reduced when a leading sound was relocated to an ipsilateral azimuth. The suppressive aftereffect was partially reduced by local application of GABA_*A*_ and glycine receptor antagonists.

### Time course and directional dependence of a sound-elicited suppressive aftereffect

A sound-elicited suppressive aftereffect in the IC similar to that revealed by the present study was reported by previous papers (Park and Pollak, 1993; Covey et al., 1996; Finlayson and Adam, 1997; Litovsky and Yin, 1998a,b; Faure et al., 2003; Mei et al., 2006; Nelson et al., 2009; Singheiser et al., 2012). Despite similarities, quantitative differences existed among different studies in the time course of suppression. The time course revealed by the present study tended to be longer than those found by some other studies (e.g., Park and Pollak, 1993; Covey et al., 1996).

Factors causing differences in the time course of suppression likely include the type of acoustic field in which a sound is presented and parameters of the sound. When a sound is presented in a free field (e.g., the present study), both ears are stimulated which leads to the activation of neurons in all structures that provide inputs to the IC. These source structures include those driven by the contralateral ear only and those driven by both ears (Cant, 2005; Schofield, 2005). When a sound is presented only to the ear contralateral to the IC in a closed field (most previous studies), it can also activate neurons in both types of structures. However, activities of neurons in binaurally driven structures can be different from those elicited by the sound presented in a free field. Thus, inputs to the IC and responses of neurons in the structure can be different under the two conditions. Previous results have demonstrated that an ipsilaterally presented leading sound can reduce the firing elicited by a contralaterally presented trailing sound in some IC neurons (Zhang and Kelly, 2009). These results support that a sound presented in a free acoustic field (e.g., the present study) generates a stronger suppressive aftereffect than when the sound is presented in a closed field to the contralateral ear alone. Other factors causing differences between our and other studies in the time course of suppression likely include acoustic parameters such as the level (Faure et al., 2003; Nelson et al., 2009) and the duration of a sound (Faure et al., 2003).

The time course of a suppressive aftereffect may also be dependent on the type of neurophysiological signal that is used to reflect the aftereffect. In the present study, LFPs generated by a population of neurons rather than action potential discharges generated by single neurons were registered. It is generally believed that the generation of an LFP depends on current dipoles produced nearby a recording electrode, which primarily reflect postsynaptic potentials created by neurons (Mitzdorf, 1985; Logothetis, 2003; Burkard et al., 2006; Goense and Logothetis, 2008). It is conceivable that a disparity exists between suppressive aftereffects reflected by the amplitude of LFP and by the strength of action potential firing, as differences exist between neurophysiological processes underlying these signals. Differences in morphological/neurophysiological/biophysical properties exist among IC neurons. These differences may lead to dissimilarities among the neurons when they generate current dipoles. The contribution of dipoles generated by different neurons to an LFP is dependent on the spatial relationship between the neurons and a recording electrode. These factors along with variations in the site of recording across different animals can lead to differences among the animals in the waveform of LFP and consequently in the suppressive aftereffect reflected by the electric potential.

Among the major findings of the present study was the directional dependence of the suppressive aftereffect generated by a sound. Such an effect was the strongest when a sound was presented at the ear contralateral to the IC (Figures 4–6). This result agrees with findings from previous studies showing that in most individual IC neurons a sound produced the strongest suppressive aftereffect when it was presented at locations where it could elicit the strongest excitatory responses over its duration (Litovsky, 1998; Litovsky and Yin, 1998a,b). For most IC neurons, these locations were at or near c90° (Litovsky and Yin, 1998b; Kuwada et al., 2011; Chot et al., 2019, 2020). Such space tuning explains results from the present study showing that a sound elicited the largest LFP as well as the strongest suppressive aftereffect when it was presented at c90°.

The peak latency of an LFP elicited by a trailing sound was not substantially affected by the temporal and spatial relationship between a leading and a trailing sound. This might be because the latency was primarily dependent on the excitatory inputs that drove the LFP response (e.g., those from the contralateral cochlear nucleus) (Cant, 2005). Latencies of such inputs may not be substantially affected by a leading sound.

### Mechanisms responsible for the sound-elicited suppressive aftereffect in the IC

Local application of Gabazine or strychnine reduced the suppressive aftereffect produced by a leading sound (Figures 7–10). During application of Gabazine, a reduction was observed only when the sound was presented at the contralateral ear. During application of strychnine, the reduction appeared to be stronger when the sound was at the contralateral than at the ipsilateral ear. Thus, both local GABA- and glycinergic inhibition contributed to the suppressive aftereffect produced by a sound in the IC in a direction-dependent manner.

GABAergic innervations received by the IC include those from the ipsilateral SPN, which is driven by excitatory inputs from the contralateral cochlear nucleus (Schofield, 1995; Kulesza and Berrebi, 2000; Saldaña et al., 2009; Felix et al., 2017). This pathway along with offset patterns of firing displayed by SPN neurons allows the stimulation of the contralateral ear to produce a suppressive aftereffect on IC neurons (Kulesza et al., 2003; Kadner and Berrebi, 2008; Gai, 2016; Salimi et al., 2017). Our results obtained during application of Gabazine (Figures 7, 8) agree with these facts. Structures providing GABAergic inputs to the IC also include the ipsilateral DNLL and VNLL as well as the contralateral DNLL (Vater et al., 1992; González-Hernández et al., 1996; Zhang et al., 1998). Each of these source structures is driven by the ear on its opposite side (Markovitz and Pollak, 1994; Wu, 1999; Batra and Fitzpatrick, 2002; Zhang and Kelly, 2006). Additionally, the DNLL is inhibited by stimulation of the ear on its same side (Markovitz and Pollak, 1994). The lack of effect of Gabazine on the aftereffect produced by a sound presented at the ear ipsilateral to the IC (Figures 7, 8) suggests that GABAergic inputs from the contralateral DNLL were not heavily involved in generating the aftereffect. It is likely that GABAergic inputs from the ipsilateral DNLL and VNLL were also not greatly involved, as synaptic responses elicited by these inputs presumably have similar time courses as those elicited by inputs from the contralateral DNLL. In contrast to the SPN, the DNLL and VNLL lack neurons with offset firing which might have prevented them from contributing to a long-lasting suppressive aftereffect in the IC. Our results regarding the function of the DNLL in generating a suppressive aftereffect seem to be in contrast with findings obtained from the Mexican free-tailed bat (Burger and Pollak, 2001) and the Mongolian gerbil (Pecka et al., 2007). These findings suggest that inputs from the DNLL can suppress responses in the IC for tens of milliseconds. Further studies have yet to be conducted to find factors that cause such a contrast.

Major glycinergic inputs to the IC are from the ipsilateral LSO and VNLL (Saint Marie et al., 1989; Yavuzoglu et al., 2011). Neurons in the LSO are excited by stimulation of the ear on the same side of the neurons while neurons in the VNLL are excited by stimulation of the ear on the opposite side (Park et al., 2004; Zhang and Kelly, 2006). Neurons in the LSO are inhibited by stimulation of the ear on the opposite side. Thus, glycinergic inputs to the IC can be activated regardless of the direction of a sound. Existing results from brain slice studies indicate that inhibitory postsynaptic potentials mediated by glycine receptors in the IC do not last longer than potentials mediated by GABA_*A*_ receptors (Moore and Trussell, 2017). If these synaptic events contributed to a suppressive effect by directly counteracting excitatory postsynaptic events, the effect would not last long and the time course of suppression observed in the present study would not have been affected by block of glycine receptors. Thus, the effect of strychnine observed in the present study (Figures 9, 10) likely suggests that complex (e.g., reverberating) local circuits that were influenced by glycinergic inputs might have been involved in the generation of the suppressive aftereffect.

The effect of a pharmacological agent on an LFP is dependent on both the area over which the agent spreads and the area within which neurons contribute to the LFP. Currently, there are no effective methods that can be used to evaluate the sizes of these areas. Some existing results from the IC suggest that a drug released microiontophoretically can reach neurons that are a few hundred micrometers away (Burger and Pollak, 1998). Thus, in the present study a pharmacological agent likely affected at least a large percentage (if not all) of the neurons that contributed to an LFP. This likelihood is supported by our results showing that amplitudes of LFPs elicited by leading and trailing sounds were both substantially increased by a drug no matter whether these sounds were presented alone or in a pair (Figures 7, 9). Despite large effects of Gabazine and strychnine on amplitudes of LFPs elicited by leading and trailing sounds, the drugs only mildly changed the suppressive effect caused by a leading sound (Figures 7–10). This result tended to suggest that mechanisms other than local inhibition had contributed to the aftereffect.

Previous findings indicate that a sound can produce suppressive aftereffects in lower auditory brain structures including the 8th nerve fibers and brainstem nuclei (Harris and Dallos, 1979; Kaltenbach et al., 1993; Finlayson and Adam, 1997). Such effects can be inherited by neurons in the IC through ascending pathways. Local mechanisms such as adaptation of excitatory response can also lead to a sound-elicited suppressive aftereffect in the IC (Singheiser et al., 2012). The contribution of adaptation is supported by the fact that for most IC neurons a sound produced the strongest suppressive aftereffect when the sound was presented at a location where it could elicit the strongest excitatory responses over its duration (Litovsky, 1998; Litovsky and Yin, 1998a,b). For most neurons in the structure, relocating a sound from c90° to i90° reduces the firing of these neurons (Chot et al., 2019, 2020). This could have lowered the degree of adaptation caused by the sound and reduced the degree of suppression of the response to a subsequent sound in the present study.

### Sound-elicited suppressive aftereffect in the IC and auditory functions

Previous studies based on activities of individual neurons have suggested that a sound-elicited suppressive aftereffect in the IC is involved in generating hearing phenomena such as forward masking (Finlayson, 1999; Faure et al., 2003; Furukawa et al., 2005; Nelson et al., 2009) and the precedence effect (Litovsky and Yin, 1998a,b; Litovsky and Delgutte, 2002; Tollin et al., 2004). Results from the present study based on activities of neural ensembles further support such involvement. One strong piece of evidence is the resemblance between the time course of suppression revealed by our study and those revealed by previous studies. The reduction of suppression caused by the relocation of a leading sound from the contralateral to the ipsilateral ear indicates that the IC is also involved in generating phenomena such as spatial release from masking. LFPs recorded in the present study did not provide information about action potential discharges in individual neurons and the threshold for eliciting these discharges. However, they provided important information related to excitatory/inhibitory interactions that underlay a suppressive aftereffect. The effect of local blockage of GABA_A_ and glycine receptors on the suppressive effect indicates that local inhibitory neurotransmission contributes to the suppressive aftereffect displayed by neural responses in the IC. It implies that such neurotransmission contributes to behavioral/psychoacoustic phenomena of forward masking and the precedence effect.

A sound-elicited suppressive aftereffect may also contribute to other aspects of spatial hearing. To understand how the detection of a recurring sound is affected by another independently recurring sound, we previously used two tone bursts to create a train of stimuli with a random order (Chot et al., 2019, 2020). Stimuli in the train were presented at a constant rate (250 ms per stimulus). Such a train could be used to mimic a “novel sound” (10% probability) interleaved with a “standard sound” (90% probability) (Chot et al., 2020). It was found that the detection of a “novel sound” by IC neurons was suppressed by a colocalized “standard sound” and the suppression was released when the two sounds were spatially separated. Results from the present study suggest that the suppression caused by a “standard sound” could be attributed to the aftereffect produced by the sound. Each presentation of a “novel sound” in a train had a high probability to be preceded by multiple presentations of a “standard sound.” A relatively short inter-stimulus interval (250 ms) enabled aftereffects produced by multiple presentations of the “standard sound” to be temporally summated to generate a large effect to suppress the response to a presentation of “novel sound” that followed. The suppressive aftereffects could be reduced when the “standard sound” was relocated from c90° to i90°. Such a change could reduce the suppression of the responses to a “novel sound” and lead to a separation-dependent enhancement in the neural detection of the sound.

In general, a sound-elicited suppressive aftereffect on neural responses in the IC is important for hearing in a natural acoustic environment in which qualitatively different sounds occurs at different time and locations. Local neural mechanisms including excitatory/inhibitory interactions contribute to the generation of such an aftereffect.

## Data availability statement

The raw data supporting the conclusions of this article will be made available by the authors, without undue reservation.

## Ethics statement

This animal study was reviewed and approved by University of Windsor Animal Care Committee.

## Author contributions

HZ conceived and designed the experiments. SA, ST, NR, and OS performed the experiments. SA and HZ analyzed the data and contributed to the writing of the manuscript. All authors contributed to the article and approved the submitted version.
